# The Six Challenges for Personality, Intelligence, Cognitive Skills, and Life Outcomes Research: An Introduction to the Special Issue

**DOI:** 10.3390/jintelligence12030035

**Published:** 2024-03-18

**Authors:** Konrad Kulikowski, Yoav Ganzach

**Affiliations:** 1 Institute of Management, Faculty of Organization and Management, Lodz University of Technology, 93-005 Lodz, Poland; 2 School of Management and Economics, The Academic College of Tel Aviv Yaffo and Faculty of Management, Tel Aviv University, Tel Aviv 69978, Israel; yoavgn@tauex.tau.ac.il

## 1. Introduction

Understanding how personality (e.g., DeNeve and Cooper 1998; Steel et al. 2019) and intellectual abilities (e.g., Gottfredson 1997, 2004b; Brown et al. 2021; Kulikowski 2021) contribute to shaping aspects of individuals’ lives is essential for in the advancement of many scientific disciplines such as psychology, management and medicine. However, although personality and intelligence and their impacts on life outcomes have been a subject of extensive research and interest among scholars across different disciplines, personality and intellectual abilities are most often studied in separation. When delving into the research on important life outcomes such as educational outcomes, career success, interpersonal relationships, mental health and overall well-being, it is vital to recognize that it is the joint influence of personality and intelligence that determines life outcomes (see, e.g., Deary et al. 2010; Damian et al. 2015; Cheng and Furnham 2012). Solely focusing on either intelligence or personality in isolation creates an artificial situation (Judge et al. 1999; Roberts et al. 2007), and only by considering them in tandem can we avoid oversimplifications and gain a deeper understanding of the processes that influence human lives (for more on the importance of ability–personality integration, see also Colom et al. (2019)).

Therefore, this Special Issue serves a dual purpose. Firstly, we want to give a platform to papers that investigate the relationships between personality, intelligence and a wide range of life outcomes. Secondly, we aim to stimulate scholarly discourse by illuminating the often neglected and underexplored topic of the simultaneous effects of intelligence and personality in shaping individuals’ life trajectories. With this editorial, our objective is to highlight the main challenges that should be addressed to facilitate the research on the effects of intelligence and personality on life outcomes and offer a comprehensive overview of potential avenues for future exploration. We hope to inspire further research on personality and intelligence effects on important life outcomes.

## 2. Challenge 1: Processes

It seems that the majority of research on predicting life outcomes from intelligence and personality is atheoretical. Although studies in this area typically document relevant associations, they usually do not explain or test the mechanisms behind these associations. Rather, they primarily show that, in statistical terms, personality and intelligence predict important life outcomes without explaining the processes underlying these associations. However, to further contribute to our understanding of the interrelations between intelligence, personality and important life outcomes, it is crucial to establish a theoretical network of the mechanism by which intelligence and personality affect life outcomes (see Aguinis and Cronin 2022). In particular, there are three types of processes which are important to the understanding of the relationship between intelligence, personality and life outcomes. First, intelligence and personality can independently affect life outcomes. This was the focus of many studies, including the recent debate between Zisman and Ganzach (Zisman and Ganzach 2022; Ganzach and Zisman 2022) and the Chicago group led by Noble Laureate in Economics Jame Heckman (Borghans et al. 2016; Golsteyn et al. 2022; see also Heckman (2011) and Heckman et al. (2013) for earlier publications) who focused on the relative predictive power of personality versus intelligence (see O’Connell (2023) for a similar focus the current Special Issue).

Second, personality and intelligence may mediate the effects of each other on life outcomes (Zhang and Ziegler 2016). An interesting example is presented in the current Special Issue in Cao and Liu’s (Cao and Liu 2023) paper that documents how depression mediates the effect of time use (i.e., working on homework, playing sports, surfing the Internet, watching TV and sleeping) on cognitive achievement tests. Earlier examples include Chamorro-Premuzic and Furnham (2008), who showed that the effect of intelligence on academic achievement was mediated by personality, or Stephan et al. (2022), who showed that personality mediates the effect of early-life intelligence on later-life cognition. We note, however, that evidence for processes in which intelligence and personality mediate the effect of each other on life outcome is rather rare in the literature, and in the more commonly encountered process research, intelligence and personality play the role of two separate predictors (e.g., Roberts et al. 2007).

Third, a most meaningful area in developing our theoretical understanding of the effects of intelligence and personality on important life outcomes lies in studying their possible moderating effects. For example, Gignac and Zajenkowski (2023) have demonstrated that a higher level of intelligence can potentially mitigate the development of a particular type of narcissistic rivalry, characterized by devaluing others, desiring their failure and displaying aggression. Moreover, Perkins and Corr (2006) found a negative relationship between performance and neuroticism, but only among individuals with lower cognitive abilities, whereas among individuals with higher intelligence, neuroticism was not significantly related to performance. Similarly, Postlethwaite et al. (2009) suggest that individuals with a high level of cognitive ability exhibit heightened safety behaviors, irrespective of their conscientiousness level, whereas conscientiousness had a more pronounced influence on safety behavior among individuals with lower levels of cognitive ability. More generally, it is not just that intelligence and personality may moderate the effect of each other on important life outcomes, but many other individual differences can be seen to moderate the impacts of these two traits. Factors such as age, culture, socioeconomic status, values and situational circumstances can all play a role. For instance, the significance of intelligence and personality might differ under extremely adverse conditions like war, poverty or starvation, as opposed to optimal life circumstances. Furthermore, the influence of intelligence and personality can vary over time and across different stages of development. Intellectual abilities may be particularly critical during certain career phases (Ganzach 2011), while personality traits may become more significant in others. An interesting example is observed among leaders in various fields such as business, culture and politics. Many individuals in contemporary societies reach the pinnacle of their careers at a rather advanced age, when cognitive abilities may not be at their peak levels. However, they still manage to fulfil their responsibilities effectively, underscoring the importance of personality in their achievements. It is also possible that the effects of personality and intelligence on life outcomes are not necessarily linear but might be curvilinear. For example, Antonakis et al. (2017) have shown that the positive perception of leaders as a function of their intelligence followed a curvilinear inverted-U function, where the best perception a leader is when their intelligence is about 1.2 standard deviations above the mean for their group of followers.

All these considerations highlight the possible dynamic nature of the effects of intelligence and personality and emphasize the need to for future researchers to ask not only whether personality or intelligence is more important for life outcomes but also when and how intelligence and personality become more influential. To sum up, we need a deeper theoretical understanding of the processes by which intelligence and personality influence our lives. 

## 3. Challenge 2: Reciprocal Relationships

Although most of the research about the relationship between intelligence/personality and life outcomes has focused on intelligence and personality as predictors and on life outcomes as dependent variables, we also need to consider to what extent life outcomes might influence personality and intelligence. Although intelligence and personality are usually treated as stable individual characteristics, there might be non-obvious reverse causality effects between them. Life outcomes such as social status, job complexity and poverty might affect intelligence and personality. This challenge is similar to the challenge we face when studying the relationship between intelligence/personality and socioeconomic success. Thus, although low intelligence is a most potent determinant of the likelihood of being below the poverty line (Herrnstein and Murray 1994), it is also possible that poverty has an important effect on one’s success in intelligence tests (Mani et al. 2013). Similarly, although cognitive ability has an important influence on job complexity (Gottfredson 1997; Wilk et al. 1995), it is also possible that the complexity of a job also influences cognitive functioning (Smart et al. 2014; Parker et al. 2021). In addition, although emotional stability and extraversion have positive effects on socioeconomic success (Seibert and Kraimer 2001), it is also possible that success increases emotional stability and extraversion (Hirschi et al. 2021). In the domain of school achievement, students’ personality traits (e.g., openness and conscientiousness) predict the use of deeper learning approaches, but learning approaches also predict personality traits. Similarly, learning approaches predict students’ grades, but grades might also influence whether students perceive learning as valuable and what approach (e.g., deep or surface-level) they apply to the learning process (Zhang and Ziegler 2018). In addition, similar reciprocal causation might possibly be encountered when studying the relationship between political ideology and intelligence (Ganzach and Schul 2021; Jedinger and Burger 2022), the relationship between political ideology and personality (Fatke 2017) or the relationship between health and intelligence (Gottfredson 2004a). Thus, by concentrating on unravelling these complex reciprocal dynamics, we can gain a more nuanced understanding of the interplay between intelligence, personality and life outcomes.

## 4. Challenge 3: What Are “Important Life Outcomes”?

An essential aspect of researching the effects of intelligence and personality on important life outcomes lies in the definition and conceptualization of “important life outcomes” itself. Although research in this area has primarily been concerned with socioeconomic success and has relied on variables such as income or educational attainment as dependent variables, there have been quite a few papers about the relationship between these variables and other life outcomes such as happiness (DeNeve and Cooper 1998; Nikolaev and Salahodjaev 2016), religiosity (Kanazawa 2010), risk taking (Gladden et al. 2009) or marital success (Gonzaga et al. 2010). The current Special Issue also provides rich and illuminating examples of life outcomes which are not often studied in intelligence and personality research, such as anti-social behavior (O’Connell (2023), which also studies risky behavior), alcohol consumption, smoking and physical activity (Li et al. 2023), as well as scientific creativity (McGregor and Frodsham 2023).

In principle, the perception of what constitutes important life outcomes may vary among individuals and cultures. Furthermore, the effects of intelligence and personality may differ when considering subjective versus objective important life outcomes. For instance, the impact on objective career success criteria such as income, occupational prestige and education level may differ from the effects on more subjective success criteria such as life or job satisfaction, happiness, sense of meaning in life and mental health. 

Moreover, how we define important life outcomes in terms of maximum performance versus typical performance (see Sackett et al. 1988; Klehe and Anderson 2007) can also influence the conclusions drawn from research on intelligence and personality. For instance, life outcomes that require maximum performance (e.g., “working smart” needs peak performance but only on occasion) may depend more heavily on intelligence, whereas outcomes that rely on typical performance (e.g., “working hard” needs average performance but maintained for a long time) may more heavily depend on personality factors such as persistence, self-esteem or optimism. 

Finally, in examining important life outcomes, it is important to pay attention to when these life outcomes were measured (Judge et al. 2010). Were they measured in the middle of the 20th century, the late 20th century or the 21st century? Were they measured in young adulthood or in later life? Thus, Ganzach and Pazy (2015) showed that the effect of intelligence, but not the effect of personality, on socioeconomic success increases with age (Ganzach and Pazy 2015), whereas the article of Marks (2023) in the current Special Issue shows that timing is important, as the effect of intelligence on income is different (stronger) in later than earlier cohorts. Thus, timing may be important in understanding the effect of intelligence and personality on life outcomes. 

## 5. Challenge 4: Conceptualization and Measurement of Intelligence and Personality

The conceptualization and measurement of intelligence and personality might influence our understanding of their effects on important life outcomes, as both of these constructs might be measured and defined in various ways. This challenge is articulated by Stankov (2023) in the current Special Issue.

### 5.1. Intelligence

Intelligence might be conceptualized and measured in different ways across different studies. It can be conceptualized as general intelligence, fluid intelligence or crystallized intelligence and assessed through achievement tests or identified within specific cognitive domains like executive attention or working memory. For example, some researchers may focus on measuring fluid intelligence, which is just one aspect of general intelligence, but still refer to it as a measure of general intelligence (Gignac 2015). This suggests that different researchers using the same terms, such as “intelligence” or “cognitive ability”, may in fact be investigating different constructs. In addition to the challenges in defining and measuring intelligence, the context in which intelligence tests are conducted can also play a significant role. Situational factors, such as the motivation to perform well on an intelligence test, can influence scores on intelligence tests (Duckworth et al. 2011; Bates and Gignac 2022). This raises the question of whether intelligence tests administered in high-stakes situations, such as in army recruitment or job interviews, might better predict important life outcomes compared to tests conducted in low-stakes situations, such as academic research on volunteers or students, where participants may not be as motivated to perform at their highest possible level.

### 5.2. Personality 

The conceptualization and measurement of personality are even more challenging than the conceptualization and measurement of intelligence. Unlike intelligence, personality is not a unitary construct. Whereas the basic conceptualization of intelligence comprises a single underlying concept—general mental ability—which underlies the relevant observables, personality is a multi-faceted concept. There is consequently less uncertainty regarding how intelligence should be defined than how personality should be defined in research about the prediction of life outcomes. In fact, most personality research pertinent to the prediction of significant life outcomes focuses on a small number of traits, each of which describes a particular aspect of personality (for examples, see Duckworth et al. (2011), for grit and Ludwig et al. (2019) for playfulness), rather than on a multi-faceted conceptualization of personality. 

To overcome this problem, scholars have often relied on the big-five personality traits (https://dictionary.apa.org/big-five-personality-model; accessed on: 13 January 2024), which, despite represents individual traits, are still considered to be a full and systematic description of one’s personality when considered together. However, one might ask, what about personality beyond the big five? There are not only different models of personality, such as HEXACO (Ashton and Lee 2009), which includes six main dimensions, but also many aspects of psychological functioning that might be included in the notion of personality such as hope, efficacy, resilience or optimism forming psychological capital (Youssef-Morgan and Luthans 2015), self-esteem (Pyszczynski et al. 2004), self-efficacy (Scholz et al. 2002) or temperamental dimensions (Langelaan et al. 2006). How should these aspects be incorporated into a comprehensive evaluation of the effect of personality on important life outcomes? Stankov (2018, 2023) refers to this question, arguing for the inclusion of additional personality dispositions in any attempt to predict important life outcome.

Another concern relates to the techniques by which we measure personality, many of which are based on self-descriptions. Is it correct to rely solely on self-descriptive measures of personality, or should we consider measures based on more objective criteria, such as judgments from others, performance assessments or records of actual behaviors? In the case of cognitive abilities, there is only a moderate association between what individuals report about their abilities and their actual performance in more objective settings (Freund and Kasten 2012). Could a similar effect be possible in personality, where we may not be fully aware of our personality traits, and they only manifest themselves in real-life situations? Can self-assessments of personality be considered a reliable measure of one’s true personality? Just as intelligence is assessed through performance tests, is it possible to create performance-based tests for personality by observing behavior in real-life situations, making them more comparable to intelligence tests? Additionally, the personality measurement methods used in different studies may differ in terms of reliability and validity. There are various approaches to measurement, including validated psychometric measures, experimental surveys, short surveys or even non-validated business measures. Measurement reliability is closely linked to predictive validity, and thus personality measures with low reliability present a significant challenge when it comes to making predictions based on personality. Yet another methodological question arises regarding structure in personality measurement: should we analyze each personality trait in separation or employ different personality traits as indicators of a common latent construct, the general factor of personality (Van Der Linden et al. 2016), similarly to the general factor of intelligence? Alternatively, should we analyze personality traits not in isolation nor as common latent factors but as combinations of traits, where certain trait patterns, such as high extraversion and conscientiousness, are more favorable than others, like high extraversion but low conscientiousness?

All these questions suggest that to achieve clarity in research and arrive at common conclusions, it may be necessary to establish more standardized methods of measuring intelligence and personality.

## 6. Challenge 5: Range Restriction

Range restriction (https://dictionary.apa.org/restriction-of-range, accessed on: 13 January 2024) refers to whether the research group accurately represents the range of variation in the variable of interest in the larger population of interest or includes a subgroup with artificially limited variation in intelligence or personality. This may be particularly important when the predictive power of intelligence is compared to the predictive power of personality and restriction on intelligence is different from the restriction on personality. In many contexts (e.g., when predicting college students’ occupational success), the range of intelligence may be constrained more than the range of personality. As a result, the correlation between intelligence and outcomes in this group might be lower, not because the “true” correlation is low, but because of the low variability in intelligence. This may lead to the impression that to understand and promote educational and economic success, we should focus more on noncognitive individual differences rather than cognitive individual differences, particularly intelligence. However, when the entire population is examined, the importance of intelligence appears to be far stronger than the importance of personality (see, for example, Zisman and Ganzach (2021) for a comparison of the predictive power of intelligence to the predictive power of grit). Thus, considering the impact of range restriction on correlation coefficients is crucial for obtaining accurate findings (Sackett et al. 2008), and using samples that are representative of the entire relevant population of interest becomes essential in exploring the true effects of personality and intelligence on life outcomes.

## 7. Challenge 6: Overlap between Personality and Intelligence

The final methodological challenge in the study of the relationship between personality/intelligence and important life outcomes is the independence of these two constructs (see, for example, Ackerman and Heggestad 1997; Carretta and Ree 2018; Major et al. 2014; Rammstedt et al. 2018) The relationship between predictors and an outcome may be altered if the predictors are correlated. In this regard, several studies have demonstrated that certain personality traits may be associated with intelligence. For example, cognitive ability is positively related to openness and emotional stability (Rammstedt et al. 2016; Anglim et al. 2022). Furthermore, personality traits may play a role in the development of intellectual skills (e.g., Ackerman 1996, PPIK model). For example, Von Stumm and Ackerman (2013) suggest that personality traits called investment traits might determine how people invest their resources as time and effort in the development of intellect. They show that, among others, openness to experience (such as readiness to re-examine traditional beliefs) or school success (such as putting a high value on educational achievement) were positively correlated with crystallized intelligence (Von Stumm and Ackerman 2013; see also Von Stumm 2013). Moreover, Ziegler et al. (2012) suggest the Openness–Fluid–Crystallized–Intelligence (OFCI) model, which hypothesizes via the environmental enrichment hypothesis that the personality trait of openness to experience has a positive effect on fluid intelligence, and this is because people with higher levels of openness tend to come across more opportunities for new learning experiences, which in turn positively impacts the development of fluid intelligence (see also Ziegler et al. 2015, 2018). Thus, this potential overlap, that is, the correlations between intelligence and personality, presents an additional challenge for researching their simultaneous predictive effects on important life outcomes.

## 8. Conclusions

In this paper, we call for more research on the joint impact of intelligence and personality on important life outcomes. We highlight six contemporary challenges for this line of research. Challenge 1 relates to the processes by which intelligence and personality influence life outcomes. We have quite a substantial body of evidence that intelligence and personality predict these outcomes, but we still know little about why and how they influence the course of people’s lives or about the possible mediating and moderating variables that might influence this process. More theoretical developments are required that will allow us to move beyond statistical reasoning in terms of the weight of correlations and regressions and allow us to understand why we observed these associations. Challenge 2 relates to reciprocal relationships between intelligence, personality and life outcomes, suggesting that not only intelligence and personality might shape trajectories of our lives but also life circumstances might impact personality and intelligence, but these reverse effects are often omitted in contemporary research. Challenge 3 concerns the definition of “important life outcomes”. These outcomes might be seen in different ways by different people in different cultures or regions of the world. What might be a success for one person might not be for another and happiness means different things for different people. Thus, it is vital to establish a set of common indicators of important life outcomes. Challenge 4 reflects a similar issue with the conceptualization and measurement of intelligence and personality. Debates about the dimensionality and measurement of both concepts are ongoing. If we do not introduce standardization to this field of research, at least in the area focusing on important life outcomes, it might become impossible to draw generalizable conclusions from contemporary research. Different measures and conceptualizations of intelligence or personality used in different studies mean that under the same common labels this research captures different phenomena. Challenge 5 refers to range restriction, an issue that has to do with the biased representation of the general population of interest, by the research sample, leading to a biased estimation of the predictive power of intelligence or personality in the general population. This challenge highlights the need for large-scale representative samples in research on the relationship between intelligence and personality, and important life outcomes. Challenge 6 relates to the possible overlap between personality and intelligence. This may be due to an overlap in the definition of the two (e.g., the definition of the personality trait of openness to experience captures aspects of intelligence), or the reciprocal influence they exert on each other during development. 

In conclusion, we hope that this Special Issue will spur further debate on the roles of personality and intelligence in forming important life outcomes. Moreover, by shedding light on the six challenges surrounding this inquiry, we hope to foster a critical examination of these complexities and inspire new and fruitful lines of research. By accounting for the challenges discussed in this editorial, we can gain new insights and minimize the risk of generating misleading conclusions when searching for fresh perspectives on the psychological processes that influence human lives.

## References

[B1-jintelligence-12-00035] Ackerman Phillip L. (1996). A theory of adult intellectual development: Process, personality, interests, and knowledge. Intelligence.

[B2-jintelligence-12-00035] Ackerman Phillip L., Heggestad Eric D. (1997). Intelligence, personality, and interests: Evidence for overlapping traits. Psychological Bulletin.

[B3-jintelligence-12-00035] Aguinis Herman, Cronin Matthew A. (2022). It’s the theory, stupid. Organizational Psychology Review.

[B4-jintelligence-12-00035] Anglim Jeromy, Dunlop Patrick D., Wee Serena, Horwood Sharon, Wood Joshua K., Marty Andrew (2022). Personality and intelligence: A meta-analysis. Psychological Bulletin.

[B5-jintelligence-12-00035] Antonakis John, House Robert J., Simonton Dean Keith (2017). Can super smart leaders suffer from too much of a good thing? The curvilinear effect of intelligence on perceived leadership behavior. Journal of Applied Psychology.

[B6-jintelligence-12-00035] Ashton Michael, Lee Kibeom (2009). The HEXACO-60: A short measure of the major dimensions of personality. Journal of Personality Assessment.

[B7-jintelligence-12-00035] Bates Timothy C., Gignac Gilles E. (2022). Effort impacts IQ test scores in a minor way: A multi-study investigation with healthy adult volunteers. Intelligence.

[B8-jintelligence-12-00035] Borghans Lex, Golsteyn Bart HH, Heckman James J., Humphries John Eric (2016). What grades and achievement tests measure. Proceedings of the National Academy of Sciences.

[B9-jintelligence-12-00035] Brown Matt I., Wai Jonathan, Chabris Christopher F. (2021). Can you ever be too smart for your own good? Comparing linear and nonlinear effects of cognitive ability on life outcomes. Perspectives on Psychological Science.

[B10-jintelligence-12-00035] Cao Xiaojie, Liu Xinqiao (2023). Time use and cognitive achievement among adolescents in China: Depression symptoms as mediators. Journal of Intelligence.

[B11-jintelligence-12-00035] Carretta Thomas R., Ree Malcolm James (2018). The relations between cognitive ability and personality: Convergent results across measures. International Journal of Selection and Assessment.

[B12-jintelligence-12-00035] Chamorro-Premuzic Tomas, Furnham Adrian (2008). Personality, intelligence and approaches to learning as predictors of academic performance. Personality and Individual Differences.

[B13-jintelligence-12-00035] Cheng Helen, Furnham Adrian (2012). Childhood cognitive ability, education, and personality traits predict attainment in adult occupational prestige over 17 years. Journal of Vocational Behavior.

[B14-jintelligence-12-00035] Colom Roberto, Bensch Doreen, Horstmann Kai T., Wehner Caroline, Ziegler Matthias (2019). Special issue “the ability–personality integration”. Journal of Intelligence.

[B15-jintelligence-12-00035] Damian Rodica Ioana, Su Rong, Shanahan Michael, Trautwein Ulrich, Roberts Brent W. (2015). Can personality traits and intelligence compensate for background disadvantage? Predicting status attainment in adulthood. Journal of Personality and Social Psychology.

[B16-jintelligence-12-00035] Deary Ian J., Weiss Alexander, Batty G. David (2010). Intelligence and personality as predictors of illness and death: How researchers in differential psychology and chronic disease epidemiology are collaborating to understand and address health inequalities. Psychological Science in the Public Interest.

[B17-jintelligence-12-00035] DeNeve Kristina M., Cooper Harris (1998). The happy personality: A meta-analysis of 137 personality traits and subjective well-being. Psychological Bulletin.

[B18-jintelligence-12-00035] Duckworth Angela Lee, Quinn Patrick D., Lynam Donald R., Loeber Rolf, Stouthamer-Loeber Magda (2011). Role of test motivation in intelligence testing. Proceedings of the National Academy of Sciences of the United States of America.

[B19-jintelligence-12-00035] Fatke Matthias (2017). Personality traits and political ideology: A first global assessment. Political Psychology.

[B20-jintelligence-12-00035] Freund Philipp Alexander, Kasten Nadine (2012). How smart do you think you are? A meta-analysis on the validity of self-estimates of cognitive ability. Psychological Bulletin.

[B21-jintelligence-12-00035] Ganzach Yoav (2011). A dynamic analysis of the effects of intelligence and socioeconomic background on job-market success. Intelligence.

[B22-jintelligence-12-00035] Ganzach Yoav, Pazy Asya (2015). Cognitive versus non-cognitive individual differences and the dynamics of career success. Applied Psychology.

[B23-jintelligence-12-00035] Ganzach Yoav, Zisman Chen (2022). Achievement tests and the importance of intelligence and personality in predicting life outcomes. Intelligence.

[B24-jintelligence-12-00035] Ganzach Yoav, Schul Yaacov (2021). Partisan ideological attitudes: Liberals are tolerant; the intelligent are intolerant. Journal of Personality and Social Psychology.

[B25-jintelligence-12-00035] Gignac Gilles E. (2015). Raven’s is not a pure measure of general intelligence: Implications for g factor theory and the brief measurement of g. Intelligence.

[B26-jintelligence-12-00035] Gignac Gilles E., Zajenkowski Marcin (2023). Intelligent grandiose narcissists are less likely to exhibit narcissistic rivalry. Personality and Individual Differences.

[B27-jintelligence-12-00035] Gladden Paul Robert, Figueredo Aurelio José, Jacobs W. Jake (2009). Life history strategy, psychopathic attitudes, personality, and general intelligence. Personality and Individual Differences.

[B28-jintelligence-12-00035] Golsteyn Bart H. H., Heckman James J., Humphries John Eric (2022). Comment on “The claim that personality is more important than intelligence in predicting important life outcomes has been greatly exaggerated”. Intelligence.

[B29-jintelligence-12-00035] Gonzaga Gian C., Carter Steve, Buckwalter J. Galen (2010). Assortative mating, convergence, and satisfaction in married couples. Personal Relationships.

[B30-jintelligence-12-00035] Gottfredson Linda S. (1997). Why g matters: The complexity of everyday life. Intelligence.

[B31-jintelligence-12-00035] Gottfredson Linda S. (2004a). Intelligence: Is it the epidemiologists’ elusive “fundamental cause” of social class inequalities in health?. Journal of Personality and Social Psychology.

[B32-jintelligence-12-00035] Gottfredson Linda S. (2004b). Life, death, and intelligence. Journal of Cognitive Education and Psychology.

[B33-jintelligence-12-00035] Heckman James (2011). Integrating Personality Psychology into Economics.

[B34-jintelligence-12-00035] Heckman James, Pinto Rodrigo, Savelyev Peter (2013). Understanding the mechanisms through which an influential early childhood program boosted adult outcomes. American Economic Review.

[B35-jintelligence-12-00035] Herrnstein Richard J., Murray Charles A. (1994). The Bell Curve: Intelligence and Class Structure in American Life.

[B36-jintelligence-12-00035] Hirschi Andreas, Johnston Claire S., Fruyt Filip De, Ghetta Anja, Orth Ulrich (2021). Does success change people? Examining objective career success as a precursor for personality development. Journal of Vocational Behavior.

[B37-jintelligence-12-00035] Jedinger Alexander, Burger Axel M. (2022). Do smarter people have more conservative economic attitudes? Assessing the relationship between cognitive ability and economic ideology. Personality and Social Psychology Bulletin.

[B38-jintelligence-12-00035] Judge Timothy A., Higgins Chad A., Thoresen Carl J., Barrick Murray R. (1999). The big five personality traits, general mental ability, and career success across the life span. Personnel Psychology.

[B39-jintelligence-12-00035] Judge Timothy A., Klinger Ryan L., Simon Lauren S. (2010). Time is on my side: Time, general mental ability, human capital, and extrinsic career success. Journal of Applied Psychology.

[B40-jintelligence-12-00035] Kanazawa Satoshi (2010). Why liberals and atheists are more intelligent. Social Psychology Quarterly.

[B41-jintelligence-12-00035] Klehe Ute-Christine, Anderson Neil (2007). Working hard and working smart: Motivation and ability during typical and maximum performance. Journal of Applied Psychology.

[B42-jintelligence-12-00035] Kulikowski Konrad (2021). Cognitive abilities: A new direction in burnout research. European Journal of Work and Organizational Psychology.

[B43-jintelligence-12-00035] Langelaan Saar, Bakker Arnold B., Doornen Lorenz J. P. Van, Schaufeli Wilmar B. (2006). Burnout and work engagement: Do individual differences make a difference?. Personality and Individual Differences.

[B44-jintelligence-12-00035] Li Hansen, Zhang Xing, Zhang Xinyue, Wang Zhenhuan, Feng Siyuan, Zhang Guodong (2023). Can intelligence affect alcohol-, smoking-, and physical activity-related behaviors? A Mendelian randomization study. Journal of Intelligence.

[B45-jintelligence-12-00035] Ludwig Rita M., Srivastava Sanjay, Berkman Elliot T. (2019). Predicting exercise with a personality facet: Planfulness and goal achievement. Psychological Science.

[B46-jintelligence-12-00035] Major Jason T., Johnson Wendy, Deary Ian J. (2014). Linear and nonlinear associations between general intelligence and personality in Project TALENT. Journal of Personality and Social Psychology.

[B47-jintelligence-12-00035] Mani Anandi, Mullainathan Sendhil, Shafir Eldar, Zhao Jiaying (2013). Poverty impedes cognitive function. Science.

[B48-jintelligence-12-00035] Marks Gary Neil (2023). Has cognitive ability become more important for education and the labor market? A comparison of the Project Talent and NLSY79 Cohorts. Journal of Intelligence.

[B49-jintelligence-12-00035] McGregor Debra, Frodsham Sarah (2023). Scientific Intelligence: Recognising it to nurture it. Journal of Intelligence.

[B50-jintelligence-12-00035] Nikolaev Boris, Salahodjaev Raufhon (2016). The role of intelligence in the distribution of national happiness. Intelligence.

[B51-jintelligence-12-00035] O’Connell Michael (2023). Assessing patterns of anti-social and risky behaviour in the Millennium Cohort Study: What are the roles of SES (socio-economic status), cognitive ability and personality?. Behavioral Sciences.

[B52-jintelligence-12-00035] Parker Sharon K., Ward M.K., Fisher Gwenith G. (2021). Can high-quality jobs help workers learn new tricks? A multidisciplinary review of work design for cognition. Academy of Management Annals.

[B53-jintelligence-12-00035] Perkins Adam M., Corr Philip J. (2006). Cognitive ability as a buffer to neuroticism: Churchill’s secret weapon?. Personality and Individual Differences.

[B54-jintelligence-12-00035] Postlethwaite Ben, Robbins Steve, Rickerson Jill, McKinniss Tamera (2009). The moderation of conscientiousness by cognitive ability when predicting workplace safety behavior. Personality and Individual Differences.

[B55-jintelligence-12-00035] Pyszczynski Tom, Greenberg Jeff, Solomon Sheldon, Arndt Jamie, Schimel Jeff (2004). Why do people need self-esteem? A theoretical and empirical teview. Psychological Bulletin.

[B56-jintelligence-12-00035] Rammstedt Beatrice, Lechner Clemens, Danner Daniel (2018). Relationships between personality and cognitive ability: A facet-level analysis. Journal of Intelligence.

[B57-jintelligence-12-00035] Rammstedt Beatrice, Danner Daniel, Martin Silke (2016). The association between personality and cognitive ability: Going beyond simple effects. Journal of Research in Personality.

[B58-jintelligence-12-00035] Roberts Brent W., Kuncel Nathan R., Shiner Rebecca, Caspi Avshalom, Goldberg Lewis R. (2007). The power of personality: The comparative validity of personality traits, socioeconomic status, and cognitive ability for predicting important life outcomes. Perspectives on Psychological Science.

[B59-jintelligence-12-00035] Sackett Paul R., Borneman Matthew J., Connelly Brian S. (2008). High stakes testing in higher education and employment: Appraising the evidence for validity and fairness. American Psychologist.

[B60-jintelligence-12-00035] Sackett Paul R., Zedeck Sheldon, Fogli Larry (1988). Relations between measures of typical and maximum job performance. Journal of Applied Psychology.

[B61-jintelligence-12-00035] Scholz Urte, Doña Benicio Gutiérrez, Sud Shonali, Schwarzer Ralf (2002). Is general self-efficacy a universal construct? Psychometric findings from 25 countries. European Journal of Psychological Assessment.

[B62-jintelligence-12-00035] Seibert Scott E., Kraimer Maria L. (2001). The five-factor model of personality and career success. Journal of Vocational Behavior.

[B63-jintelligence-12-00035] Smart Emily L., Gow Alan J., Deary Ian J. (2014). Occupational complexity and lifetime cognitive abilities. Neurology.

[B64-jintelligence-12-00035] Stankov Lazar (2018). Low correlations between intelligence and Big Five personality traits: Need to broaden the domain of personality. Journal of Intelligence.

[B65-jintelligence-12-00035] Stankov Lazar (2023). Intelligence, personality, and the prediction of life outcomes: Borghans et al. (2016) vs. Zisman and Ganzach (2022) debate. Journal of Intelligence.

[B66-jintelligence-12-00035] Steel Piers, Schmidt Joseph, Bosco Frank, Uggerslev Krista (2019). The effects of personality on job satisfaction and life satisfaction: A meta-analytic investigation accounting for bandwidth-fidelity and commensurability. Human Relations.

[B67-jintelligence-12-00035] Stephan Yannick, Sutin Angelina R., Luchetti Martina, Aschwanden Damaris, Terracciano Antonio (2022). IQ in adolescence and cognition over 50 years later: The mediating role of adult personality. Intelligence.

[B68-jintelligence-12-00035] Van Der Linden Dimitri, Dunkel Curtis S., Petrides K. V. (2016). The General Factor of Personality (GFP) as social effectiveness: Review of the literature. Personality and Individual Differences.

[B69-jintelligence-12-00035] Von Stumm Sophie (2013). Investment traits and intelligence in adulthood: Assessment and associations. Journal of Individual Differences.

[B70-jintelligence-12-00035] Von Stumm Sophie, Ackerman Phillip L. (2013). Investment and intellect: A review and meta-analysis. Psychological Bulletin.

[B71-jintelligence-12-00035] Wilk Steffanie L., Desmarais Laura Burris, Sackett Paul R. (1995). Gravitation to jobs commensurate with ability: Longitudinal and cross-sectional tests. Journal of Applied Psychology.

[B72-jintelligence-12-00035] Youssef-Morgan Carolyn M., Luthans Fred (2015). Psychological capital and well-being. Stress and Health: Journal of the International Society for the Investigation of Stress.

[B73-jintelligence-12-00035] Zhang Jing, Ziegler Matthias (2016). How do the big five influence scholastic performance? A big five-narrow traits model or a double mediation model. Learning and Individual Differences.

[B74-jintelligence-12-00035] Zhang Jing, Ziegler Matthias (2018). Why do personality traits predict scholastic performance? A three-wave longitudinal study. Journal of Research in Personality.

[B75-jintelligence-12-00035] Ziegler Matthias, Cengia Anja, Mussel Patrick, Gerstorf Denis (2015). Openness as a buffer against cognitive decline: The Openness-Fluid-Crystallized-Intelligence (OFCI) model applied to late adulthood. Psychology and Aging.

[B76-jintelligence-12-00035] Ziegler Matthias, Danay Erik, Heene Moritz, Asendorpf Jens, Bühner Markus (2012). Openness, fluid intelligence, and crystallized intelligence: Toward an integrative model. Journal of Research in Personality.

[B77-jintelligence-12-00035] Ziegler Matthias, Schroeter Titus, Lüdtke Oliver, Roemer Lena (2018). The enriching interplay between openness and interest: A theoretical elaboration of the OFCI model and a first empirical test. Journal of Intelligence.

[B78-jintelligence-12-00035] Zisman Chen, Ganzach Yoav (2021). In a representative sample grit has a negligible effect on educational and economic success compared to intelligence. Social Psychological and Personality Science.

[B79-jintelligence-12-00035] Zisman Chen, Ganzach Yoav (2022). The claim that personality is more important than intelligence in predicting important life outcomes has been greatly exaggerated. Intelligence.

